# Thermophilic Microbial Inoculant Promotes Lignocellulose Degradation During Green Waste Composting

**DOI:** 10.3390/microorganisms14061177

**Published:** 2026-05-23

**Authors:** Dan Hao, Xiaohang Yu, Xiangyang Sun, Dongdong Cheng, Hao Ding, Yige Wang, Yalin Li, Zhewen Geng, Guijun Xu

**Affiliations:** 1 The Key Laboratory of Comprehensive Utilization of Green Waste in Hebei Province, College of Forestry, Beijing Forestry University, Beijing 100083, China; haodan_jm@bjfu.edu.cn (D.H.);; 2 School of Economics and Management, Beijing Forestry University, Beijing 100083, China; 3 Research Institute of Sand Control and Utilization, Fuxin 123000, China

**Keywords:** green waste, thermophilic microbial inoculant, lignocellulose degradation, metagenomic analysis, core taxa

## Abstract

Thermophilic microbial inoculant (CI) has been demonstrated to optimize the green waste composting (GWC) process. The pathways through which it enhances lignocellulose degradation remain unclear. This study evaluated composting performance under four treatments: CI, effective microorganisms (EM), *Phanerochaete chrysosporium* (WF), and natural composting (CK). To elucidate the biological differences between efficient lignocellulose-degrading systems and CK, metagenomic analyses were conducted on CI and CK based on lignocellulose degradation rates. The results indicated that CI inoculation did not negatively affect the compost heating process and produced a nitrogen-rich, safe, and mature compost product. Compared to other treatments, CI increased the lignocellulose degradation rate by 3.66% to 31.8%. Metagenomic analysis revealed that CI inoculation enriched genes encoding glycoside hydrolases (GHs), glycosyl transferases (GTs), carbohydrate esterases (CEs), and carbohydrate-binding modules (CBMs) across multiple composting phases, positively impacting dominant carbohydrate-active enzyme (CAZyme) families including AA3, CE1, and CE7. CI inoculation also elevated the relative abundance of lignocellulose-degrading microorganisms (0.70~2.73%), simplified microbial network structure, and strengthened microbial cooperation. Within the microbial network, *Chryseolinea*, *Protaetiibacter*, and unclassified_f__Burkholderiaceae were identified as core taxa involved in lignocellulose degradation. Redundancy analysis (RDA) identified temperature as the primary factor influencing biological factors, with CI improving composting efficiency by optimizing the microenvironment. Collectively, this work provides a novel strategy for microbial inoculant application in composting and offers new perspectives for identifying core taxa, contributing to advancing composting efficiency.

## 1. Introduction

Composting represents a promising approach for recycling green waste (GW) [1]. It utilizes microorganisms and their enzymes to transform unstable organic matter into stable humus-like substances, yielding compost that enhances urban soil fertility and plant quality [2]. However, the high lignocellulose content in GW limits microbial decomposition and utilization [3]. Moreover, GW rich in branches and trunks contains elevated lignin levels, conferring greater resistance to microbial degradation and reducing conversion efficiency [4,5]. Advancing the capacity of lignin-degrading microorganisms is therefore crucial for efficient, high-quality GW recycling.

Microbial inoculation increases the abundance and activity of functional microorganisms and their secreted enzymes, accelerating the degradation of complex organic matter, including lignocellulose [6]. The thermophilic phase is critical for lignin degradation during composting, as high temperatures stimulate microbial metabolism, which is essential for the breakdown of complex organic matter [7,8]. Consequently, thermophilic microorganisms have become valuable resources for developing microbial inoculants. Sun et al. [9] optimized composting by inoculating the thermophilic bacterium *Caldibacillus thermoamylovorans*, accelerating the temperature rise and humification process while reducing greenhouse gas emissions. Wan et al. [10] found that lignin-degrading fungi could promote directed humification during composting, enhancing compost quality. Sequential inoculation of both mesophilic and thermophilic fungi further improved efficacy. Within green waste composting (GWC) systems, lignocellulose degradation is collaboratively accomplished by fungi, bacteria, and actinobacteria [11]. Neglecting synergistic interactions among these microbial groups may impair the degradative capacity of inoculated microorganisms [12]. Therefore, to construct an efficient lignocellulose-degradation system, Wang et al. [13] inoculated a microbial consortium comprising thermophilic bacteria (*Bacillus amyloliquefaciens*), thermophilic actinobacteria (*Streptomyces thermoviolaceus*), and mesophilic fungi (*Clonostachys rogersoniana*) during the thermophilic phase of GWC. Notably, *C. rogersoniana* exhibits optimal growth at 30 °C, potentially compromising its function during the thermophilic phase. Moreover, enhanced interactions between thermophilic aerobic bacteria and thermophilic fungi significantly enhance compost quality [14]. Consequently, there is a critical gap in research on synergistic lignin degradation by thermophilic fungi, bacteria, and actinomycetes in GWC that requires further investigation. The practical effects of this inoculant, as well as its impacts on microbial community dynamics and carbohydrate-active enzymes (CAZymes) gene variations during GWC, require further elucidation.

In recent years, rapid advances in metagenomics have enabled comprehensive analysis of lignocellulose degradation mechanisms during GWC [15,16]. Through this technology, researchers have uncovered principal microbial communities and CAZymes involved in lignocellulose degradation during composting. Chen et al. [17] employed metagenomic analysis to comprehensively profile the thermophilic-phase microbial communities, functional genes, and metabolic traits of mulberry branch-cow dung composting under a synthetic microbial community (SynCom), revealing that SynCom inoculation accelerates lignocellulose degradation by restructuring the genetic potential and metabolic functions of microbial community. Lu et al. [18] utilized metagenomics to demonstrate that zero-valent iron and MnSO_4_ primarily enhance composting efficiency by increasing the relative abundance of lignocellulose-decomposing bacteria and functional genes during the thermophilic phase. Zhang et al. [19] integrated high-throughput 16S rRNA sequencing with metagenomic analysis, finding that cow dung restructures microbial community structure and network complexity, augments diversity and functional specificity, and identifies *Thermopolyspora* as a key taxon for humus formation. Furthermore, researchers have employed metagenomic analysis to identify key biological factors involved in lignocellulose degradation, with *Bacillus*, *Actinomycetota*, *Mucoromycota*, *Pseudoxanthomonas*, AA1, AA3, GH12, and CE4 postulated as critical contributors to this process during composting [15,20,21,22,23,24]. However, current studies primarily focus on agricultural waste, with limited attention to lignocellulose degradation in GWC. The microbiological mechanisms of GWC remain primarily reliant on high-throughput sequencing technologies [12,25], lacking support from CAZymes data. Furthermore, microorganisms exhibiting high connectivity with other taxa, partitioned via co-occurrence networks, are also significant for lignocellulose degradation [26]. These populations maintain network structure and function, playing a pivotal role in lignocellulose degradation [27]. However, their low relative abundance often excludes them from statistical analyses. Current research has primarily focused on dominant microbial communities and their functions, with relatively limited studies on high-connectivity nodes within microbial networks. It remains unclear how high-connectivity nodes encoding lignocellulose-degrading CAZymes influence composting efficiency, or the role of the thermophilic microbial inoculant (CI) in this process. Therefore, the inoculation of GWC systems with a synergistic consortium of thermophilic fungi, bacteria, and actinobacteria to degrade lignin offers a novel strategy for enhancing composting efficiency. Furthermore, integrating composting experiments with metagenomic analysis, CAZyme annotation, and network analysis is crucial for deciphering CAZyme and microbial community dynamics during efficient composting to improve lignocellulose degradation rates. This approach provides new insights into CAZyme-microbe linkages in GWC, shifts focus from dominant microbial taxa to high-connectivity nodes, deepens micro-scale understanding of GWC, and delivers valuable theoretical and practical guidance for developing high-efficiency composting systems.

Therefore, this study investigates the efficacy of a CI in GWC and its regulatory pathways for lignocellulose degradation. The objectives include: (1) evaluating the efficacy of a synergistic lignin-degrading consortium of thermophilic fungi, bacteria, and actinomycetes during GWC and lignocellulose degradation; (2) employing metagenomics to comprehensively analyze GWC, focusing on enzyme genes and microbial community dynamics involved in lignocellulose degradation; and (3) identifying shared, highly synergistic microbial taxa across composting phases and key factors through which CI regulates lignocellulose degradation. This work aims to advance understanding of microbial inoculants in lignocellulose degradation during GW composting, thereby contributing to improved composting efficiency.

## 2. Materials and Methods

### 2.1. Experimental Materials and Reagents

#### 2.1.1. Composting Materials

Green waste (GW) was sourced from Fragrant Hills Park (Beijing, China) comprising pruned branches of *Pinus tabuliformis*, *Robinia pseudoacacia*, *Ulmus pumila*, and *Koelreuteria paniculata* generated during summer 2022 landscape maintenance. The GW was shredded to a particle size of less than 1.5 cm using a mechanical grinder before composting. The basic parameters of the GW were as follows: moisture content 47.4%, pH 7.30, electrical conductivity (EC) 0.66 mS/cm, total organic carbon (TOC) 545 g/kg, total Kjeldahl nitrogen (TKN) 1.03%, C/N ratio 53.8, hemicellulose content (HC) 17.1%, cellulose content (CC) 31.6%, and lignin content (LC) 21.4%. Urea (high-purity grade) was commercially sourced from Shandong Hualu-Hengsheng Group Co., Ltd., Dezhou, China. The plastic film used was a high-transmittance light blue PO film, procured from Jiangsu Dinghai Farm Plastic Film Co., Ltd., Suqian, China.

#### 2.1.2. Microbial Materials

The formulation of the thermophilic microbial inoculant (CI) was derived from previous studies [28]. It included: a thermophilic fungus (*Thermomyces* sp., F1; 99.84% similarity to *T. lanuginosus* NF3^T^), a thermophilic actinobacterium (*Streptomyces* sp., A1; 99.72% similarity to *S. thermoviolaceus* NBRC 15459^T^), and two thermophilic bacteria (both *Bacillus* sp., B4 and B7, where B4 shares 99.93% similarity with *B. licheniformis* SR63^T^, and B7 shows 99.03% similarity with *B. gelatini* TMW 2.552^T^). *Phanerochaete chrysosporium* is a white-rot fungus (WF) which was purchased from the Beijing Microbiological Culture Collection Center (Beijing, China) with the original accession number NDM3-2. Effective microorganisms (EM), purchased from Foshan Nanhai Chantai Animal Pharmaceutical Co., Ltd. (Foshan, China), comprise a consortium containing *Lactobacillus* spp., actinomycetes, yeasts, *Bacillus subtilis*, *Azotobacter* spp., *Bacillus natto*, *Aspergillus* spp., halophilic bacteria, and *Kyosei* VS-34. The three microbial inoculants were selected based on their demonstrated efficacy in lignocellulose degradation within GW and prevalent application in experimental research. These strains in CI exhibit lignin-degrading potential, growing at 50 °C while decolorizing aniline blue medium, and demonstrating superior lignocellulose degradation in solid-state fermentation trials [28]. WF depolymerizes lignin by secreting extracellular enzymes, and has been shown to enhance green waste composting (GWC) efficiency, frequently serving as a control inoculant in GWC studies [25,29]. As a commercial inoculant, EM demonstrates validated efficacy in promoting cellulose and hemicellulose degradation and humification. It is also commonly used as the reference inoculant in GWC experiments [25,30].

Potato dextrose broth (PDB), commonly used for fungal cultivation, has also been demonstrated effective for growing *Bacillus* spp. and *Streptomyces* spp. strains by Su et al. [31] and Gerayeli et al. [32]. Therefore, to maintain consistency with the WF and CI, PDB was selected as the universal medium. Before composting, colonies of F1, A1, B4, B7, and white-rot fungus were inoculated into 250 mL Erlenmeyer flasks containing PDB. The inoculated flasks were then placed in a shaker at 180 rpm for cultivation. Specifically, A1, B4, and B7 were cultivated at 50 °C for 2 days, F1 at 50 °C for 5 days, and white-rot fungus at 28 °C for 5 days [28]. Following cultivation, F1, A1, B4, and B7 cultures were mixed in equal volumes to form CI.

### 2.2. Experimental Design and Composting Operations

The experiment was conducted from September to December 2022 at a composting facility in Fragrant Hills Park, Beijing, China. Four treatments were established: a blank control with natural composting (CK), two experimental controls (EM and WF), and the experimental treatment (CI). Inoculation details are in Table 1. The inoculation amount was determined based on by Du et al. [33].

Before composting, shredded GW from Section 2.1 was divided evenly into eight piles (150 kg each). Moisture content was adjusted to approximately 60% by adding water, and the initial C/N ratio to approximately 25:1 to 30:1 using urea. Each treatment was inoculated with the respective microbial inoculant according to Table 1, with two replicate piles. Inoculants were diluted with water (1:10, *v*/*v*) before application. After inoculation, materials were formed into conical heaps with a base radius of approximately 1 m. On day 5, heating phase samples were collected and piles turned. Subsequent pile turnings occurred every 6 days until day 53, when cooling phase samples were collected and turning ceased. Moisture was adjusted when necessary during turning. Liu et al. [34] demonstrated that temperatures below 10 °C prevent composting materials from entering the thermophilic phase without external additives. Therefore, when local temperatures fell near 10 °C, piles were covered with plastic film to improve heat retention [35]. These films reduced low nighttime temperature impacts on compost. Additionally, increasing the scale of the compost pile can trigger a rise in temperature, maintaining the compost at the optimal range for lignin degradation (40~50 °C) [8,36]. To extend lignin degradation, when compost entered the cooling phase and temperatures first dropped below 40 °C, the two piles from each treatment were combined into a larger pile.

The composting process lasted 88 days. Samples were collected on days 0, 5, 23, 53, and 88, representing the initial, heating, thermophilic, cooling, and maturation phases of composting, respectively. A multi-point method collected samples from upper, middle, and lower pile sections [37]. Samples were homogenized by quartering, then placed in plastic bags and sent to the laboratory. In the laboratory, each sample was divided into three portions: one was stored at −80 °C, another at 4 °C, and the remainder was shade-dried, then ground, sieved, and stored. Before combining the piles, two piles were established per treatment, with one sample collected from each pile. Then, two equal mass samples were gathered from the two piles and combined to form a new sample, resulting in a total of three samples. After combining the piles, each treatment was represented by one pile, from which three samples were collected.

### 2.3. Measurement and Calculation of Parameters

The pile temperatures were measured at noon on days 0, 1, 2, 3, 4, 5, 7, 9, 11, 13, 15, 17, 19, 21, 23, 26, 29, 32, 35, 38, 41, 44, 47, 50, 53, 60, 67, 74, 81, and 88. During measurement, the thermometer was inserted into the front, middle, rear, and bottom of the pile (at a depth of at least 30 cm), and readings were recorded after stabilization [36,38]. Each pile’s temperature was calculated as the average of these four measurements. The temperature of mature compost was used as a reference. This compost was a pile of GW that had completed the composting process. Using the composting pile’s temperature as a reference bypasses ambient temperature increases induced by the greenhouse warming, allowing for a better judgment of the composting maturation phase. The composting process was considered complete when the temperature of each treatment pile decreased to the level of the mature compost. The pH, EC, TOC, TKN, and ash content of the composting materials were determined according to the methods described by Yang and Zhang [36]. Moisture content was determined following the methods described by Yu et al. [30]. The contents of lignocellulose components (lignin, cellulose, or hemicellulose) were determined following the methods described by Yang et al. [39]. Specifically, LC was calculated as the difference between acid detergent lignin (ADL) and ash content; CC was determined as the difference between acid detergent fiber (ADF) and ADL; and HC was derived as the difference between neutral detergent fiber (NDF) and ADF.

The lignin degradation rate (LDR), cellulose degradation rate (CDR), and hemicellulose degradation rate (HDR) were calculated based on the principle of ash conservation [40] and using Equation (1).

(1) $$\mathrm{Degradation}\;\mathrm{rate}\;(\%)=(1-\frac{X_{1}M_{2}}{X_{2}M_{1}})\times 100\%.$$

Here, *X*_1_ and *X*_2_ represent the initial and final ash contents, respectively, while *M*_1_ and *M*_2_ denote the initial and final contents of lignocellulose components (lignin, cellulose, or hemicellulose), respectively.

### 2.4. Metagenomic Sequencing and Data Analysis

As a natural composting system, CK exhibits dynamics of microorganisms and carbohydrate-active enzymes (CAZymes) that are both representative and universal. Section 3.2 results indicate that CI demonstrates the highest lignocellulose degradation rate compared to EM, WF, and CK, signifying its optimal degradation efficiency in this study. Investigating the underlying micro-scale changes will contribute to a deeper understanding of effective lignocellulose degradation processes. Based on these findings, this study focused exclusively on the CK and CI to elucidate the pathways underlying the thermophilic microbial inoculant of lignocellulose degradation in GWC systems. Analyzed compost samples included: initial phase (Initial), CK heating phase (CK_H), CI heating phase (CI_H), CK thermophilic phase (CK_T), CI thermophilic phase (CI_T), CK cooling phase (CK_C), CI cooling phase (CI_C), CK maturation phase (CK_M), and CI maturation phase (CI_M). Three replicates per treatment group yielded 27 total samples.

Total genomic DNA extraction from compost microbial communities, metagenomic sequencing, and annotation and abundance profiling of microorganisms and carbohydrate-active enzymes (CAZymes) were conducted by Majorbio Bio-Pharm Technology Co., Ltd. (Shanghai, China). DNA was extracted with the E.Z.N.A.^®^ Soil DNA Kit (Omega Bio-tek, Norcross, GA, USA), and metagenomic sequencing was performed on the Illumina NovaSeq 6000 platform (San Diego, CA, USA). Microbial annotation and abundance profiling used Diamond (v2.0.13) alignment against the nonredundant (NR) database (v20230830). CAZyme annotation employed HMMER (v3.1b2) against the CAZy database (v8.0). All metagenomic data processing utilized the Majorbio Cloud Platform (www.majorbio.com). Based on CAZymes analysis results from Section 3.3.2, we reconstructed a lignocellulose-degrading microbial gene set using the original gene set. This set was aligned against the NR database (v20230830) using Diamond (v2.0.13) for species annotation, functional annotation, and abundance information. CAZyme abundances were normalized to RPKM (reads per kilobase of transcript per million mapped reads) on the cloud platform. The microbial analysis during the composting process focused solely on bacteria and fungi.

### 2.5. Network Analysis

Based on the results of Spearman correlation analysis and Benjamini–Hochberg correction [41], nodes exhibiting *p* < 0.05 and |r| > 0.9 were used to construct bacterial–fungal co-occurrence network (BFGN) and lignocellulose-degrading microorganism network (LMGN) at the genus level. LMGN subnetworks were extracted using the subgraph function from the R package “igraph” (v2.1.4), selecting microorganisms with relative abundance > 1 × 10^−9^ as representatives [42]. Topological properties, network robustness, within-module connectivity (Zi), and among-module connectivity (Pi) were computed for both the global network and subnetworks using the R package “igraph”(v2.1.4). Network complexity was calculated following the method provided by Yao et al. [43]. Given the complexity of lignocellulose degradation and the distinct aspects of node importance captured by different topological properties, high-connectivity nodes were identified using multiple approaches: (1) based on Zi/Pi values [43], nodes designated as network hubs (Zi > 2.5, Pi > 0.62), module hubs (Zi > 2.5, Pi ≤ 0.62), or connectors (Zi ≤ 2.5, Pi > 0.62); (2) based on eigenvector centrality [44], nodes exhibiting high eigenvector centrality (>95th percentile). All networks were visualized with Gephi (v0.11.0-SNAPSHOT).

### 2.6. Identification of Core Taxa

Core taxa in GWC systems were identified through integrated metagenomic sequencing, co-occurrence network analysis, random forest analysis, and Spearman correlation analysis [45,46,47]. The criteria for inclusion in this core taxa were as follows: (1) the ability to encode CAZymes referenced in Section 3.3.2; (2) classification as high-connectivity nodes within the network analysis, present in the LMGN and all its subnetworks; and (3) a statistically significant contribution to HC, CC, and LC (*p* < 0.01), with consistent results across both random forest and correlation analyses. These criteria ensure both the association of the selected taxa with lignocellulose degradation and their persistently high connectivity across composting environments. Details of network analysis follow Section 2.5.

Random forest analysis used the R package “randomForest” (v4.7-1.2) with 90% of samples allocated to the training set and 10% as the test set for validation. Spearman correlations were analyzed in Origin 2023b.

### 2.7. Data Analysis

Data processing used Microsoft Excel 2019. Physicochemical parameters were statistically analyzed and visualized in Origin 2023b, while metagenomic datasets underwent analysis and visualization using the Majorbio Cloud Platform (www.majorbio.com) and RStudio (v4.4.2).

## 3. Results and Discussion

### 3.1. Characterization of Base Parameters in the Composting Process

**Figure 1:**
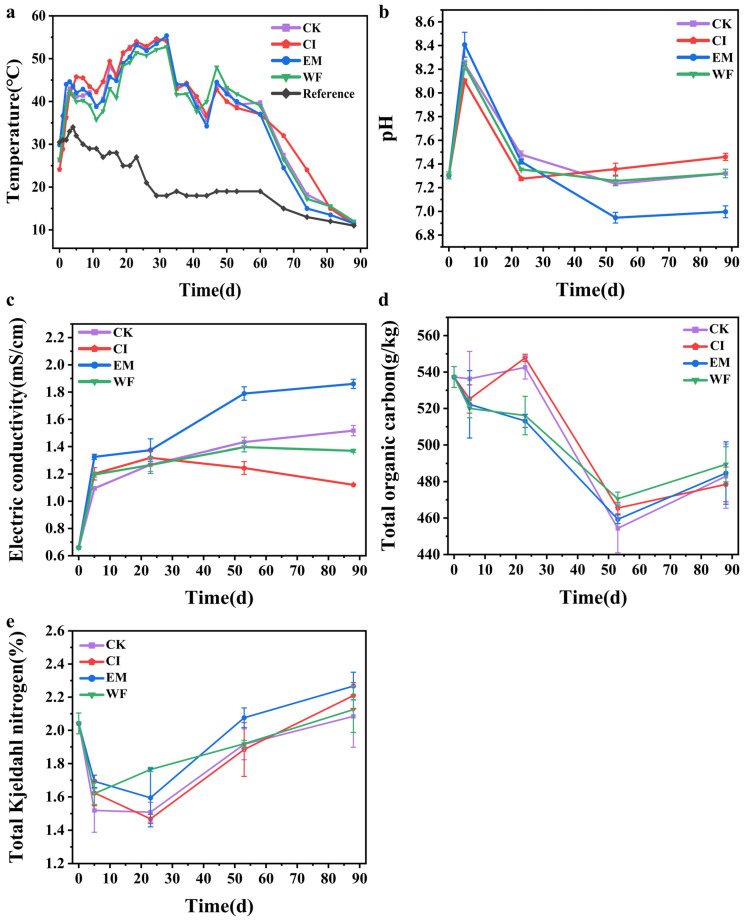
Variations in basic parameters in different treatments during green waste composting. (**a**) Temperature. (**b**) pH. (**c**) Electrical conductivity. (**d**) Total organic carbon. (**e**) Total Kjeldahl nitrogen. The error bars in the figure represent the standard deviation. Based on the results of Fisher’s protected least significant difference test, different letters represent significant differences between treatments in the same index (*p* < 0.05).

Temperature is a critical factor influencing the composting process [48]. Its variation is closely associated with the heat released during microbial proliferation [49]. Across treatments, temperatures showed similar progression patterns (Figure 1a). All treatments maintained a thermophilic phase (≥50 °C) for more than 5 days, meeting the requirements for pathogen reduction and compost safety [38,50]. The peak temperatures in the CK, CI, and EM compost piles all reached 55 °C. Notably, both the CK and CI entered the thermophilic phase earlier than EM and sustained this phase for an extended duration (an additional two days). This is likely attributable to the microorganisms of the CI, which was derived from the thermophilic phase of GWC and exhibits superior adaptability to the GWC environment [51]. During the initial phase of composting, the higher pH in EM may have impeded microbial nutrient absorption, resulting in reduced microbial activity and consequently slowing the rate of temperature increase [52]. WF exhibited the shortest thermophilic phase, a relatively slower heating rate, and a lower overall composting temperature. This phenomenon may be attributed to competition between allochthonous and indigenous microorganisms, as well as the relatively low optimal growth temperature for *Phanerochaete chrysosporium* [8,51]. Additionally, combining compost piles elevated temperatures above 40 °C, extending the period of efficient lignin degradation by microorganisms [8,53]. However, readily degradable organic matter became the primary nutrient source for microorganisms during this process [53]. Therefore, the lower temperature increase observed in CI after combining may be attributed to the extensive consumption of readily degradable organic matter early in the composting process [36,53]. Compared to traditional composting, the process extended to 88 days, likely due to the additional heating process that occurred after the piles were combined, as well as the plastic film’s thermal insulation reducing cooling rates. In summary, the addition of CI did not negatively affect the composting process.

The pH in all treatments exhibited an initial increase followed by a subsequent decline (Figure 1b). In the early composting phase, a sharp rise in pH to 8.1~8.4 was observed, driven by the release of substantial NH_4_^+^-N from rapid microorganism decomposition of nitrogenous compounds [10,30]. Subsequently, ammonia volatilization, transformation, and organic acid production enhanced H^+^ accumulation, driving pH reduction across all treatments to 7.0~7.5 [30,36,54]. Furthermore, the addition of lignin-degrading and cellulose-degrading microbial inoculant promotes the growth of ammonifying bacteria, which results in higher NH_4_^+^-N concentrations [10]. This may explain why the pH in CI remained higher than in other treatments during the cooling and maturation phases. Electrical conductivity (EC) indicates soluble salt concentrations in composting materials (Figure 1c) [55]. From day 0 to day 23, EC increased across all treatments, primarily due to inorganic salt release (e.g., phosphates, metal ions) during organic matter decomposition [30]. As composting progressed, water-soluble ions (e.g., NH_4_^+^, NO_3_^−^) participated in humification [30]. Concurrently, the solubility of some mineral salts decreases as pH rises [36]. The highest pH in CI beyond day 53 likely contributed to its significantly lower EC (*p* < 0.05) relative to other treatments. This observation aligns with the phenomenon reported by Wan et al. [10]. By the end of composting, the EC for CK, CI, EM, and WF were 1.52 mS/cm, 1.12 mS/cm, 1.86 mS/cm, and 1.37 mS/cm, respectively, all of which meet the requirements for non-toxic compost (<4 mS/cm) [38].

Carbon and nitrogen constitute essential microbial nutrients [55,56]. During GWC, total organic carbon (TOC) content exhibited an overall declining trend (Figure 1d). The period from day 23 to 53 was characterized by heightened microbial activity, during which TOC was rapidly decomposed and mineralized, releasing substantial CO_2_ [55]. Within this period, TOC content decreased by 88.2 g/kg (CK), 82.3 g/kg (CI), 53.8 g/kg (EM), and 45.6 g/kg (WF), accounting for 95.3% to 162% of the total TOC loss throughout the composting process. After day 53, the retention of organic carbon increased in all treatments. This may be associated with an increase in microbial biomass and enhanced humification processes [10,57]. At composting termination, CI exhibited the highest TOC degradation, showing 8.19 to 23.0% greater reduction than other treatments. This confirms CI inoculation significantly enhances TOC degradation in GW [58]. All treatments exhibited similar total Kjeldahl nitrogen (TKN) content dynamics: an initial decline followed by recovery (Figure 1e). During the early composting phase, intense ammonification by microorganisms and sustained thermophilic conditions inhibited nitrifying bacteria growth, triggering substantial ammonia emissions and sharp TKN reduction [59,60]. Particularly, TKN content in CI declined to 1.47%. This may be due to the higher temperatures maintained in the CI. Subsequently, organic matter mineralization generated a “concentration effect”, and microbial conversion of labile NH_4_^+^-N to stable compounds (e.g., NO_3_^−^-N and humic acids) created favorable conditions for nitrogen retention [10,36]. Consequently, after day 23, all treatments showed increasing TKN content. By the end of composting, the TKN content in CI, EM, and WF surpassed those of CK (2.08%), reaching 2.21%, 2.27%, and 2.12%, respectively. This indicates that microbial inoculation enhances nitrogen retention in composting materials, increasing the nitrogen content in the final compost product [10,29,55].

### 3.2. Changes in Lignocellulose During Composting and Final Degradation Rate

**Figure 2:**
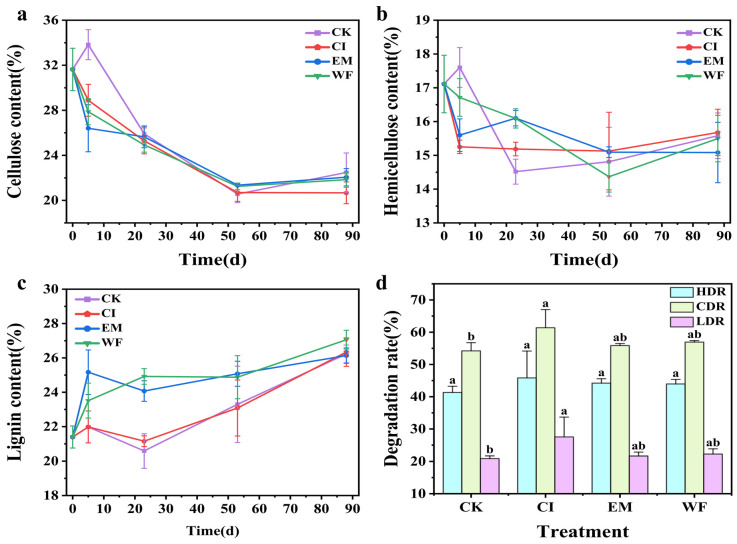
Variations in lignocellulose content in different treatments during green waste composting. (**a**) Hemicellulose content. (**b**) Cellulose content. (**c**) Lignin content. The degradation rate (**d**) of lignocellulose at the end of composting. CK: the treatments without added microbial inoculum; CI: the treatment with added thermophilic microbial inoculant; EM: the treatment with added effective microorganisms; WF: the treatment with added white-rot fungi; HDR: hemicellulose degradation rate; CDR: cellulose degradation rate; LDR: lignin degradation rate. The error bars in the figure represent the standard deviation. Based on the results of Fisher’s protected least significant difference test, different letters represent significant differences between treatments in the same index (*p* < 0.05).

Lignocellulose, an organic carbon component resistant to microbial utilization [61], resists degradation due to its complex molecular structure [62]. This property often causes a relative concentration effect during composting, where lignocellulose component concentrations may initially increase [48,62]. However, as microbes actively degrade the lignocellulose components, this effect diminishes or disappears, leading to a reduction in their concentrations. Thus, lignocellulose content variations indirectly reflect microbial consumption of different carbon sources throughout composting. Dynamic changes in lignocellulose component concentrations displayed distinct trends during the composting period (Figure 2a–c).

By the end of composting, the cellulose content (CC) in CK, CI, EM, and WF decreased by 28.9%, 34.7%, 30.3%, and 30.9%, respectively, compared to the original material. Hemicellulose content (HC) decreased to a lesser extent than CC. By the end of composting, HC declined by only 8.4%~11.9% across treatments. Lignin content (LC) showed an inverse trend to that of cellulose and hemicellulose. LC showed an overall increase of 22.1%~26.5% across all treatments by the end of composting. Cellulose was the most extensively degraded lignocellulosic component during composting. And the addition of CI enhances the microbial degradation of cellulose in GW [30,60]. Consequently, the CI exhibited a steady decline in CC throughout composting, whereas for the CK, EM, and WF, a reduction in CC was observed only during partial phases. Hemicellulose is also a major carbon source for microbial growth and metabolism [62,63]. Although all treatments exhibited HC reduction during partial phases, the HC in CI, EM, and WF exhibited a sustained decline over a longer period compared to CK. Thus, microbial inoculants facilitate enhanced hemicellulose utilization in GW [63]. Lignin is widely recognized as the most recalcitrant component of lignocellulose [60,63], and its concentration dynamics exhibit an inverse trend relative to those of cellulose and hemicellulose. In the early phase of composting, microbes rapidly consume the more readily available substrates (e.g., cellulose and hemicellulose), thereby inducing an initial rise in LC in all treatments [60,61]. As temperatures rise, the cleavage of chemical bonds within lignocellulose [63], along with the degradation of cellulose and hemicellulose and high nitrogen loss, may stimulate microbial lignin degradation [48,61]. Consequently, a decline in LC was observed in CK, CI, and EM from days 5 to 23. In contrast, the decline in LC in WF occurred later than in other treatments, observed only from days 23 to 53. This delay may be attributed to the optimal temperature range of 36~40 °C for *Phanerochaete chrysosporium* [8]. During the later stages of composting, the nutrients released from lignin degradation are insufficient to fully support microbial growth, thereby reducing degradation efficiency and resulting in a sustained increase in LC [53].

In this study, the degradation rates of lignocellulose components were calculated based on the principle of ash content conservation [40], which helps eliminate the relative concentration effects on lignocellulose. The final degradation rates of lignocellulose components are illustrated in Figure 2d. Generally, microbial inoculants stimulate biological activity, enhancing utilization of both cellulose and hemicellulose [64]. Compared with the other treatments, CI increased the lignocellulose degradation rate by 3.66% to 31.8%, indicating that the addition of CI effectively promotes lignocellulose degradation during GWC [65]. Consequently, by the end of composting, cellulose degradation rate (CDR) in the CI, EM, and WF reached 61.4%, 55.8%, and 56.9%, respectively, while hemicellulose degradation rate (HDR) was 45.8%, 44.2%, and 44.0%. All values exceeded those observed in CK (54.2% and 41.3%, respectively), with CI exhibiting a significantly higher CDR than CK (*p* < 0.05). Meanwhile, by the end of composting, the lignin degradation rate (LDR) in CI (27.5%) was higher than those in other treatment, and it was significantly greater than that in CK (20.9%, *p* < 0.05). The degradation of lignocellulose components can influence each other [53,61,66]. The CI demonstrated significantly higher degradation rates of cellulose, hemicellulose, and lignin compared to EM, WF, and CK, indicating superior degradation potential. Furthermore, lignocellulose serves as a preferred substrate for microorganisms under high-temperature conditions [67], and lignocellulose-degrading microorganisms generally favor thermophilic environments [68]. The comparatively extended thermophilic phase in CI likely provided favorable conditions for these microorganisms, resulting in enhanced lignocellulose degradation relative to EM and WF. Compared to CK, the addition of CI increased both the abundance of genes encoding lignocellulose-degrading enzymes (Section 3.3.2) and the relative abundance of relevant microbial taxa (Section 3.4.1), thereby enhancing its potential for efficient lignocellulose degradation [69].

### 3.3. Characteristics of Carbohydrate-Active Enzymes During Composting

#### 3.3.1. Carbohydrate-Active Enzyme

Carbohydrate-active enzymes (CAZymes) are essential tools for microorganisms, catalyzing the degradation, modification, and biosynthesis of carbon-containing organic matter [70]. A total of 280854 genes associated with CAZymes were identified across all compost samples. Collectively, across all composting phases, the total abundance of CAZymes genes in CI consistently exceeded that in CK, with values of 46,271.63, 45,349.65, 51,555.59 and 50,986.67 compared to 45,732.75, 44,803.12, 48,542.26 and 50,266.73, respectively. Ma et al. [71] have indicated that elevated CAZymes gene abundance enhances lignocellulose degradation. This finding suggests that the positive effect of CI addition on CAZyme gene abundance contributes to its superior lignocellulose degradation rate [69].

The CAZymes genes were annotated by metagenomic analysis in this study (Table S1). Glycoside hydrolases (GHs) primarily catalyze degradation of diverse carbohydrates by cleaving glycosidic bonds between carbohydrate and non-carbohydrate moieties [72]. GHs accounted for the highest proportion of genes, reaching 34.3%, a result that is consistent with the findings of Wang et al. [73]. Following the thermophilic phase, the abundance of GHs in CI was consistently higher than that in CK. This suggests that the addition of CI enriches GH genes, thereby enhancing the potential for organic matter degradation [74]. The GWC system was also rich in glycosyl transferase (GT) genes [75], which constituted 32.6% of the total gene count. Certain GTs families, such as GT4, also participate in the remodeling of low-molecular-weight components derived from lignocellulose degradation [71]. Consequently, GTs abundance in CI increased by 1.52% to 8.69% compared to CK, which may facilitate more rapid consumption of lignocellulose degradation products, thereby positively influencing the lignocellulose degradation process. Moreover, carbohydrate esterases (CEs) were found to constitute 15.7% of the total gene count. CEs catalyze O- and N-deacetylation of acetylated sugar residues in non-cellulosic polysaccharides (e.g., hemicellulose) and enhance the activity of hydrolytic enzymes secreted by fungi and bacteria [76]. CI increased the abundance of CE genes during the heating, cooling, and maturation phases, indicating a greater potential for hemicellulose degradation. Auxiliary activities (AAs), primarily comprising redox enzymes, participate in the degradation of plant cell wall components such as lignin and cellulose [77]. However, the proportion of AA genes in the GWC system was relatively low, accounting for only 9.25% of the total. Moreover, the effect of CI addition on AAs abundance was limited, with CI surpassing CK only during the maturation phase. Guo et al. [52] reported that AAs are the primary enzymes involved in lignin degradation during the later phases of composting. Therefore, the upregulation of AAs abundance observed in the later phases following CI addition may positively influence lignin degradation. Carbohydrate-binding modules (CBMs), though lacking catalytic activity themselves, facilitate the function of other CAZymes by enhancing accessibility and affinity between microbial enzymes and substrates [78,79]. Throughout the GWC process, CI sustained higher abundance of CBMs. Although the proportion of CBMs was relatively low at 4.80%, they play a crucial role in lignocellulose degradation [16]. Therefore, the addition of CI may enhance the enzymatic catalytic efficiency in the composting system compared to CK, thereby promoting more effective lignocellulose degradation.

#### 3.3.2. Carbohydrate-Active Enzymes Involved in Lignocellulose Degradation

Based on CAZy database annotations and previous studies, a total of 82 CAZyme families and subfamilies associated with lignocellulose degradation were identified, primarily distributed among GH, AA, CE, and CBM [21,22,66,73,79,80,81,82,83,84,85,86,87,88,89,90,91,92,93,94,95,96,97,98]. The composition and temporal dynamics of abundance of these families are illustrated in Figure S1. The cumulative abundance of CAZyme families involved in lignocellulose degradation exhibited an initial decline followed by a subsequent increase in both CK and CI (Table S2). This phenomenon may be attributed to the mortality of lignocellulose-degrading microorganisms entering the thermophilic phase, leading to a decline in their relative abundance (Section 3.4.1). Correlation analysis revealed a positive relationship between the cumulative abundance of CAZymes involved in lignocellulose degradation and CC (correlation coefficient of 0.07), and a significant positive correlation with LC and HC (correlation coefficients of 0.39 and 0.44, respectively). These findings are consistent with those reported by Zhang et al. [20]. Therefore, the higher abundance of CAZyme genes observed in CI following the thermophilic phase may promote an increased rate of lignocellulose degradation [73].

**Figure 3:**
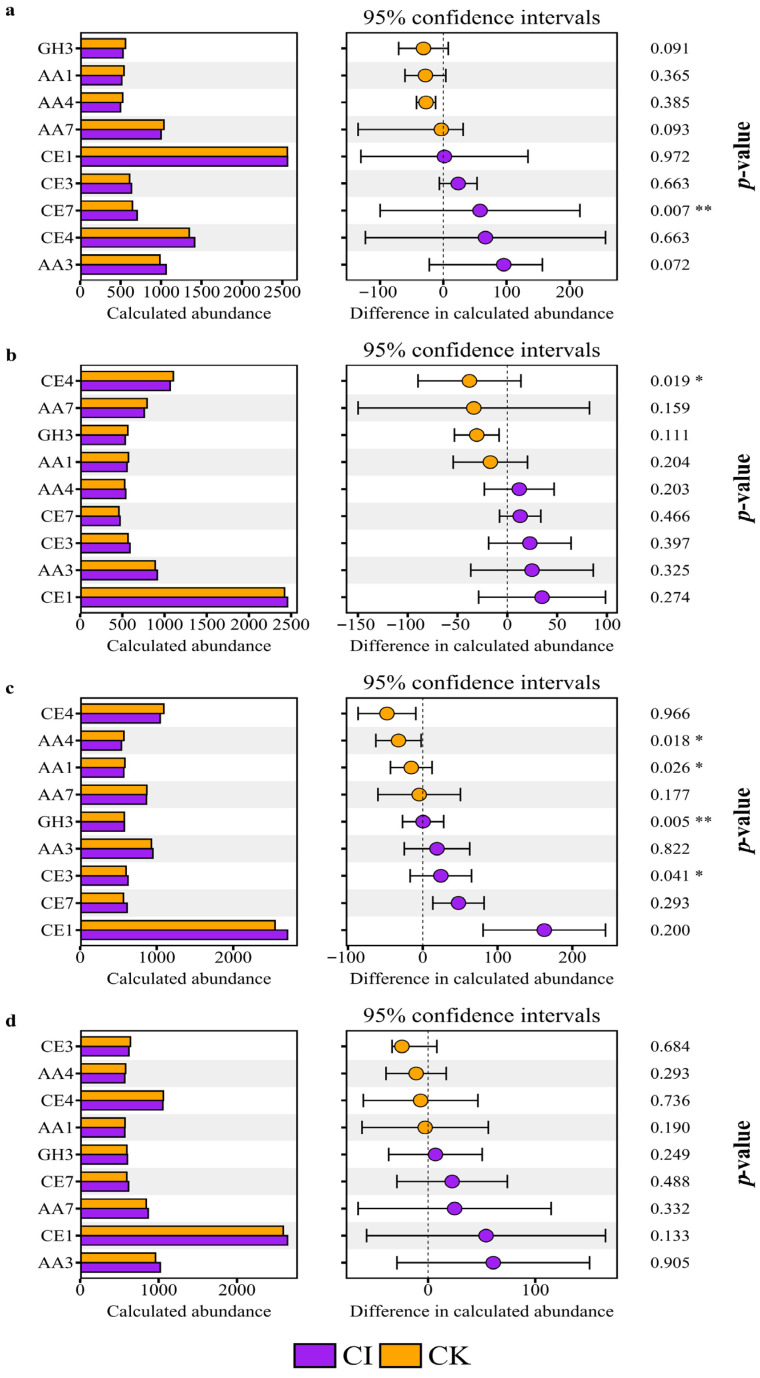
Intergroup differences in abundance between CK and CI in dominant carbohydrate-active enzymes (CAZyme) families during the (**a**) heating, (**b**) thermophilic, (**c**) cooling, and (**d**) maturation phases. CAZyme family abundance is the abundance after RPKM normalization. Data normality was assessed using the Shapiro–Wilk test. For normally distributed data, statistical differences were determined using independent sample *t*-tests; for non-normally distributed data, Wilcoxon rank-sum tests were applied (* *p* < 0.05, ** *p* < 0.01).

An analysis of the intergroup differences among the dominant CAZyme families (top nine) involved in lignocellulose degradation is presented in Figure 3a–d. Correlation analyses revealed that these dominant CAZyme families exhibited significant relationships with one or more components of lignocellulose (Figure S2). This indicates that these CAZyme families play a crucial role in the degradation of lignocellulose. Throughout the composting process, the abundances of AA3, CE1, and CE7 in CI consistently exceeded those in CK. Notably, AA3 and CE7 were significantly enriched during periods of higher HC, whereas CE1 and CE7 exhibited significant enrichment during phases of higher LC. Additionally, CE1 also showed marked enrichment when CC was relatively low. Zhang et al. [20] noted that AA3, CE1, and CE7 represent the primary CAZyme families implicated in lignocellulose degradation. These results indicate that the increased abundance of AA3, CE1, and CE7 following CI addition may enhance lignocellulose degradation. AA3 generate hydrogen peroxide as a byproduct, which subsequently participates in lignin degradation and modification via Fenton reactions [80]. CE1 facilitates lignocellulose degradation by disrupting lignin–carbohydrate complexes [97]. CE7 possesses a unique and narrow specificity towards acetylated substrates, which contributes to hemicellulose degradation [94]. Integrating these observations with Section 3.3.1 suggests CI likely modulates lignocellulose degradation in GWC through the following pathways: (1) promoting cleavage of lignin–hemicellulose linkage bonds; (2) enhancing catalytic efficiency of hydrolytic enzymes via auxiliary enzymes; (3) accelerating consumption of lignocellulose degradation products; and (4) driving preferential cellulose and hemicellulose degradation, thus enhancing subsequent lignin degradation.

### 3.4. Lignocellulose-Degrading Microorganisms in Green Waste Composting

#### 3.4.1. Lignocellulose-Degrading Microorganisms

**Figure 4:**
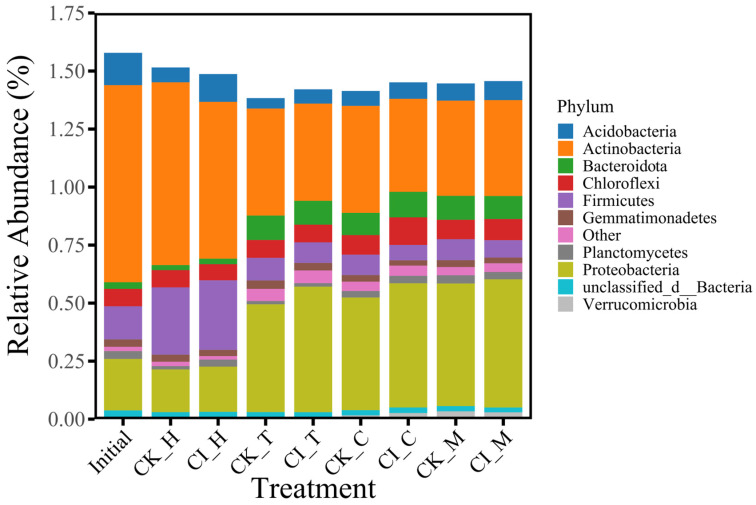
Cumulative relative abundance of lignocellulose-degrading microorganisms. Initial: initial phase; CK_H: CK heating phase; CI_H: CI heating phase; CK_T: CK thermophilic phase; CI_T: CI thermophilic phase; CK_C: CK cooling phase; CI_C: CI cooling phase; CK_M: CK maturation phase; CI_M: CI maturation phase.

The reconstruction of gene sets based on results from Section 3.3.2 identified 126 microbial phyla associated with lignocellulose degradation, whereas 99.92% were bacteria and only 0.08% fungi (Table S3). Across all samples, bacterial abundance was significantly higher than that of fungi, indicating that bacteria serve as the dominant microorganisms of lignocellulose degradation during GWC. This may be associated with the relatively low diversity of fungal communities and slower colonization efficiency, which hinders the establishment of fungal populations [99]. However, lignocellulose-degrading microorganisms represented a minor fraction within the composting system, with a cumulative relative abundance of only 1.38~1.58% (Figure 4). Compared with CK, the cumulative relative abundance of lignocellulose-degrading microorganisms in CI increased by 0.70~2.73% during the thermophilic to maturation phases. This indicates that CI addition promotes the enrichment of lignocellulose-degrading microorganisms.

Actinobacteria and Proteobacteria were the dominant microbial taxa involved in lignocellulose degradation during GWC. In the early composting phase, lignocellulose-degrading communities were dominated by Actinobacteria, with relative abundance ranging from 0.40% to 0.85%. However, Actinobacteria are k-strategy bacteria that grow rapidly only when sufficient labile substrates are available [100]. Consequently, progressive conversion of labile organic matter into stable humic-like substances during composting favored increased relative abundance of Proteobacteria (0.18~0.55%), replacing Actinobacteria as the predominant phylum by the thermophilic phase. Both Actinobacteria and Proteobacteria encode various lignocellulose-degrading genes [20,66,101], performing important functions in lignocellulose degradation. Consequently, their higher relative abundances likely facilitate lignocellulose degradation initiation. CI addition markedly elevated the relative abundance of Proteobacteria, with increases of 4.58~16.5% across composting phases. Furthermore, Proteobacteria nodes were the dominant microbial nodes in lignocellulose-degrading microorganism network (LMGN) and its subnetworks (Table S4). Moreover, although Actinobacteria exhibited lower relative abundances in CI than in CK, their nodal proportion in LMGN subnetworks increased during multiple composting phases. Integration of cumulative abundance of lignocellulose-degrading enzymes found that CI likely revealed lignocellulose degradation in GWC by upregulating degradation-associated gene expression and enhancing microbial interactions [66].

#### 3.4.2. Characteristics of the Lignocellulose-Degrading Microorganism Network

**Figure 5:**
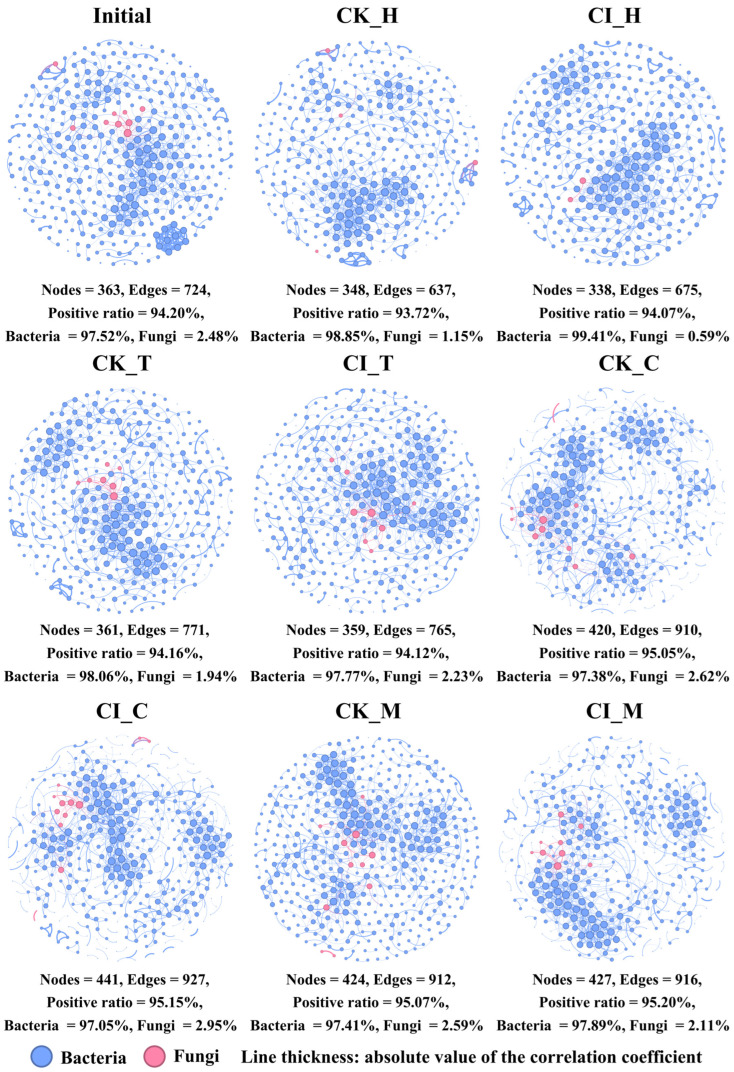
Interactions of lignocellulose-degrading microorganisms across composting phases. Initial: initial phase; CK_H: CK heating phase; CI_H: CI heating phase; CK_T: CK thermophilic phase; CI_T: CI thermophilic phase; CK_C: CK cooling phase; CI_C: CI cooling phase; CK_M: CK maturation phase; CI_M: CI maturation phase.

To assess how composting phases and CI addition influenced microbial interactions within lignocellulose-degrading microorganisms, subnetworks corresponding to CK and CI were extracted from the LMGN for each composting phase (Figure 5). Bacteria were the dominant microbial nodes across all subnetworks, comprising 97.05%~99.41% of the nodes. Network topology analysis (Table 2) indicates lower robustness, complexity, and modularity in CI, reflecting a simplified network structure [43,102]. While the enhancement of the aforementioned microbial community metrics may confer greater resilience and adaptability to environmental fluctuations [103], the relatively simplified network in CI correlates with superior fermentation quality [104]. This could be related to the reduction in redundant functional modules in microbial interactions resulting from the network simplification [105]. The comparatively simplified network structure of the LMGN, relative to the bacterial–fungal co-occurrence network (BFGN), further corroborates these findings. Conversely, CI addition increased the number of high-connectivity nodes during the thermophilic and cooling phases. These nodes reportedly mediate organic matter decomposition, and their elevated number bolsters microbial community stability [106,107]. In addition, microbial interactions have been reported as a primary driver of ecological processes [108]. Across all subnetworks, positive correlation ratios exceeded 90% with positive cohesion > 0.85 (>0.5), indicating that interactions among lignocellulose-degrading microorganisms during composting are predominantly mutualistic [108,109]. Compared to CK, CI exhibited higher positive cohesion and a greater proportion of positive edges, indicating enhanced synergistic partnerships among lignocellulose-degrading microorganisms. These interactions serve as fundamental elements of community persistence, system stability, and degradation capacity [74]. The simplified network structure coupled with enhanced synergy in CI may confer superior lignocellulose degradation performance to GWC system.

#### 3.4.3. Identification of Core Taxa of Lignocellulose Degradation

**Figure 6:**
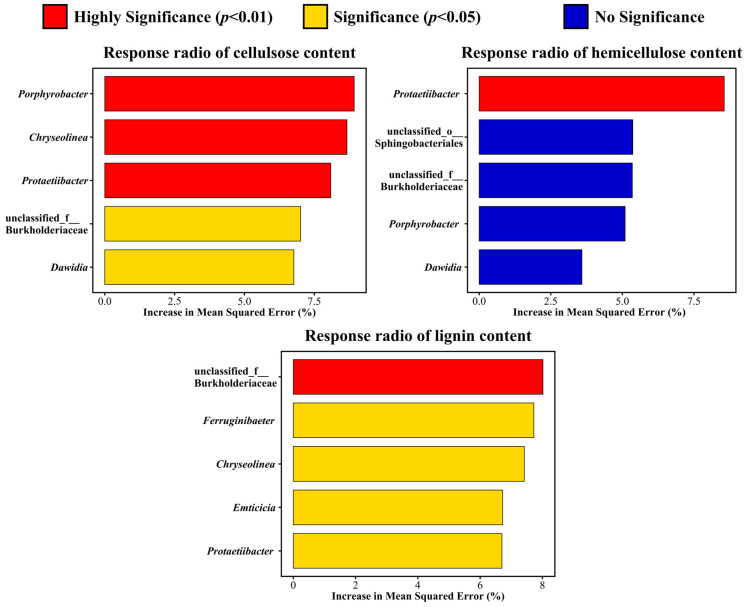
Results of random forest analysis.

A comparison of the high-connectivity node types present in the LMGN and its subnetworks revealed a total of 16 shared high-connectivity nodes, which are distributed across Proteobacteria, Bacteroidota, and Actinobacteria (Table S5). Among these, Bacteroidota accounted for the majority of nodes (68.75%), followed by Proteobacteria (25.00%), with Actinobacteria representing the smallest fraction (6.25%). To identify the primary taxa among these shared high-connectivity nodes that contribute to lignocellulose degradation, we evaluated their contributions to variations in CC, HC, and LC using random forest analysis (Figure 6). *Porphyrobacter*, *Chryseolinea*, and *Protaetiibacter* exhibited strongly significant contributions to CC variations. *Protaetiibacter* also showed strongly significant contributions to HC variations, while LC variations were predominantly influenced by unclassified_f__Burkholderiaceae.

*Chryseolinea* is a key genus of microorganisms involved in cellulose degradation and transformation [110]. Li et al. [25] reported significant enrichment of *Chryseolinea* following the addition of *Trametes* sp. during GWC, further underscoring its advantage in structural carbon degradation. The unclassified_f__Burkholderiaceae represents an uncultured taxon. However, members of Burkholderiaceae encode AA3 and AA6 genes and exhibit significant potential for lignin degradation [89]. *Protaetiibacter* has been confirmed to participate in xylose metabolism [111], but its ability or potential role in cellulose degradation remains unclear. Whether it facilitates cellulose degradation by promoting hemicellulose breakdown requires further validation. Currently, there are no reports on the role of *Porphyrobacter* in lignocellulose degradation. Nevertheless, *Porphyrobacter* can degrade polycyclic aromatic hydrocarbons, and its relative abundance significantly increased under lignin-enriched conditions [112,113]. Thus, *Porphyrobacter* is potentially a key genus for lignin degradation. The correlation results (Figure S3) indicate that all four microbial taxa exhibited significant associations with one or more components of lignocellulose, suggesting their critical roles in lignocellulose degradation [52]. Specifically, *Protaetiibacter* exhibited significant correlations with HC, CC, and LC; *Chryseolinea* correlated significantly with CC and LC; and unclassified_f__Burkholderiaceae showed a similar pattern to *Chryseolinea*. These results were consistent with the random forest analysis, and the above three microorganisms were identified as core taxa involved in lignocellulose degradation. However, *Porphyrobacter* showed a significant correlation only with HC, which is inconsistent with the results of the random forest analysis. Furthermore, existing research provides no reports of *Porphyrobacter* degrading cellulose. Therefore, this microorganism is proposed only as a candidate core taxon for lignocellulose degradation. During composting, the relative abundances of candidate core taxa in CK and CI differed across composting phases (Table S6). Compared to CK, *Chryseolinea* and unclassified_f__Burkholderiaceae exhibited higher relative abundances in CI during multiple phases, while *Protaetiibacter* showed higher relative abundance only during the cooling phase. This may be attributable to the addition of lignin-degrading microorganisms, which facilitates the enrichment of *Chryseolinea* [25], and to the enhanced lignin degradation following CI addition, which provides more phenolic compounds to recruit Burkholderiaceae [114]. Consequently, *Chryseolinea* and unclassified_f__Burkholderiaceae are inferred to be core taxa in CI. CI likely optimizes lignocellulose degradation by upregulating their abundances and modulating the microbial interactions associated with these taxa.

However, this study employed a relatively stringent correlation threshold when constructing the co-occurrence network [115,116], aiming to investigate the pathways by which strongly associated nodes and their resulting interaction networks enhance lignocellulose degradation performance. This approach inevitably favors microbial taxa with the strongest correlations, potentially overlooking the bridging or buffering roles that taxa with moderate association strengths might play in the degradation process. Consequently, our understanding of lignocellulose degradation may become overly centered on the primary framework of the network. Given the complexity of lignocellulose degradation, exploring the influence of a broader range of microbial taxa is necessary. Future studies should investigate how the identity and diversity of core taxa vary under different correlation thresholds, or even incorporate functional prediction, to provide a more comprehensive explanation of microbial contributions to lignocellulose degradation. This will offer a theoretical basis for optimizing GWC processes, including the development of novel inoculants.

### 3.5. The Relationships Between Environmental and Biological Factors

**Figure 7:**
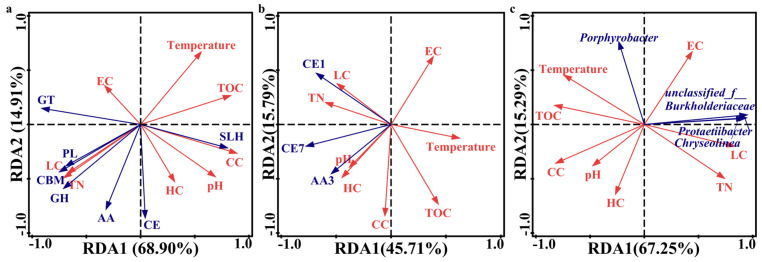
The effects of environmental factors on biological factors. The redundancy analysis results for (**a**) carbohydrate-active enzymes (CAZyme), (**b**) key CAZyme families, and (**c**) core taxa. AA: auxiliary activity; CBM: carbohydrate-binding module; CE: carbohydrate esterase; GH: glycoside hydrolase; GT: glycosyl transferase; PL: polysaccharide lyase; SLH: s-layer homologous module; EC: electrical conductivity; TOC: total organic carbon; TKN: total Kjeldahl nitrogen; HC: hemicellulose content; CC: cellulose content; LC: lignin content. CAZymes and key CAZyme family abundance is the abundance after RPKM normalization.

Redundancy analysis (RDA) elucidated relationships between environmental and biological factors (Figure 7). Environmental factors appear as red arrows, biological factors as blue arrows. The length of each environmental factor reflects the extent of its association with biological factors, with longer arrows indicating stronger relationships. Angles between vectors represent correlation direction and magnitude, quantified through cosine values. Positive and negative cosines indicate positive and negative correlations, respectively; larger absolute cosine values correspond to stronger correlations.

Table S7 presents the importance of environmental factors on biological factors. Hemicellulose is an organic material that is readily decomposed and utilized by microorganisms [99]. Its degradation relies on CAZymes secreted by microorganisms. Therefore, it exerted a significant influence (*p* < 0.05) on CAZyme. As the most extensively degraded component in GWC, cellulose exerted a highly significant influence on the dynamics of both CAZyme gene abundance and core taxa populations (*p* < 0.01). This may be related to CAZymes and microorganisms involved in cellulose degradation or the consumption of its degradation products [117]. Lignin is one of the most recalcitrant organic matters during composting. Its content fluctuations primarily exert significant influence on core taxa. During lignin degradation, increasing LC can stimulate specific functional nodes involved in aromatic compound metabolism [113]. This also explains why the abundance of most biological factors was positively correlated with LC.

During composting, temperature dynamics drive microbial community succession [11] and significantly influence the abundances of core taxa, CAZymes, and key CAZyme families. Different microorganisms exhibit varying temperature adaptations, and the enzymes they secrete possess distinct thermal tolerances. Although thermophilic microorganisms dominate lignocellulose degradation, the sharp temperature increase from the heating phase to the thermophilic phase exerts substantial stress on microbial survival. This leads to high mortality among mesophilic populations, reducing overall microbial abundance [118] and consequently affecting CAZyme abundance. pH is a primary factor shaping specific CAZyme families [119] and significantly impacts the abundances of both CAZymes and key CAZyme families. This relationship likely stems from the preferences of microorganisms harboring CAZyme genes for certain acidic or alkaline environments. Combining Section 3.1 and Section 3.4.1, CI addition resulted in lower pH levels being maintained under higher temperatures, potentially creating a more conducive environment for lignocellulose-degrading microorganisms. This could positively influence the enrichment of genes encoding CBMs, GHs, etc. Conversely, for AAs, which participate in lignocellulose degradation during the later composting phases, CI elevated the pH during these phases to promote the enrichment of AA genes, thereby facilitating more effective lignocellulose degradation. Furthermore, TOC and EC significantly impacted the abundance of key CAZyme families, while TN significantly influenced core microorganisms. Carbon, nitrogen, and EC likely affect the microbial community by altering the levels of nutrients essential for microbial survival [120,121].

Collectively, temperature emerges as the predominant factor influencing biological factors. However, different environmental factors exert distinct effects on different biological factors. Consequently, the impact of temperature on composting performance can be modulated by optimizing environmental factors. For instance, during the development of microbial inoculants, it is crucial to consider the environmental changes induced by the addition of these inoculants, as well as their implications for lignocellulose degradation. Integrating these effects into the assessment framework provides better regulatory strategies for enhancing composting efficiency.

From a macro perspective, the pilot-scale experiments demonstrated the practical efficacy of a synergistic degradation system comprising thermophilic fungi, thermophilic bacteria, and thermophilic actinomycetes within the GWC, thereby offering a novel strategy to enhance composting efficiency. Focusing on CAZymes as the central axis, the analysis of lignocellulose degradation-associated enzyme genes and microorganisms offers new micro-level insights into GWC, while further clarifying the biological factors involved in lignocellulose degradation during composting. Compared to examining the overall composting microbiome, co-occurrence network analysis specifically targeting lignocellulose-degrading microorganisms more effectively reveals differences in interactions within specific functional microbial communities under natural composting versus CI-amended conditions. Furthermore, the integrated approach combining CAZymes, microorganisms, and co-occurrence networks provides a new methodology for identifying specific functional taxa, with random forest and correlation analyses further confirming the significance of these taxa in lignocellulose biodegradation. This multi-level analytical framework, integrating both macro and micro perspectives, enables a more comprehensive understanding of the changes in lignocellulose degradation and the dynamics of associated biological factors during composting. It establishes a novel approach for identifying candidate crucial biological factors in complex organic matter degradation processes and provides a novel perspective for applying metagenomics to investigate the GWC, contributing to the advancement of recycling systems for GW and promoting environmental sustainability.

However, we acknowledge that the roles of core taxa and dominant CAZyme families remain inferred, necessitating direct functional validation to elucidate their mechanistic contributions. Furthermore, low-abundance CAZyme families in composting deserve attention. Future research should employ extensive sampling integrated with techniques capable of pinpointing active microorganisms and enzymes (e.g., metatranscriptomics and qPCR), following the analytical framework of this study, to identify the active biological factors involved in lignocellulose degradation within GWC systems and to provide deeper insights into the biological mechanisms of the composting process. Furthermore, controlling carbon emissions during composting has emerged as a current research focus, with lignocellulose constituting a major carbon-containing organic matter in GWC. While CI addition enhances lignocellulose degradation, whether it reduces greenhouse gas emissions remains unclear. Future studies could conduct composting trials in closed-system reactors to verify the efficacy of CI in greenhouse gas mitigation.

## 4. Conclusions

This study investigated the effects of CI on the GWC process and lignocellulose degradation. The results show that CI exhibited no adverse impact on the composting process and demonstrated optimal efficacy in promoting lignocellulose degradation, increasing the degradation rate by 3.66% to 31.8%. The addition of CI also increased the cumulative abundance of CAZyme families and microbial taxa associated with lignocellulose degradation (notably Proteobacteria). The addition of CI also enhanced the interactions among lignocellulose-degrading microorganisms, simplifying the LMGN structure and enhancing the stability of microbial communities during both the thermophilic and cooling phases. Additionally, CI enriched AA3, CE1, and CE7 genes and promoted the growth of core taxa (e.g., *Chryseolinea* and unclassified_f__Burkholderiaceae) by modulating the composting microenvironment, thereby optimizing the composting process. These findings demonstrate CI’s potential for improving GW resource recovery and provide a novel framework for targeted identification of core functional taxa involved in lignocellulose degradation during GWC. Future studies could integrate core taxa identification with targeted inoculation strategies to explore shared lignocellulose-degrading core taxa across diverse composting amendments, elucidate their functional dynamics under varying composting conditions, and establish targeted theoretical and practical foundations for high-efficiency composting technologies.

## Figures and Tables

**Table 1:** Inoculation details for different treatments.

Treatment	Inoculum Information	Inoculation Amount (%, *v*/*w*, Dry Weight Basis)
CK	NA	0
CI	Thermophilic microbial inoculant	1.0
EM	Effective microorganisms	1.0
WF	*Phanerochaete chrysosporium*	1.0

“NA” indicates no microbial inoculum were added. Effective microorganisms: a commercial inoculant.

**Table 2:** Topological properties of microbial co-occurrence networks.

Network Name	Network Complexity	Positive Cohesion	Negative Cohesion	Modularity	Robustness
BFGN	0.78	0.91	−0.31	0.67	18.01
LMGN	0.10	0.91	−0.08	0.80	3.92
Initial	0.29	0.86	−0.11	0.73	3.93
CK_H	0.26	0.86	−0.11	0.73	3.60
CI_H	0.15	0.88	−0.11	0.72	3.93
CK_T	0.30	0.86	−0.12	0.73	4.21
CI_T	0.29	0.86	−0.12	0.72	4.20
CK_C	0.08	0.89	−0.10	0.75	4.27
CI_C	0.07	0.90	−0.10	0.76	4.16
CK_M	0.08	0.89	−0.10	0.75	4.25
CI_M	0.08	0.90	−0.10	0.76	4.23

BFGN: bacterial–fungal co-occurrence network; LMGN: lignocellulose-degrading microorganism network. All remaining networks represent LMGN subnetworks: Initial: initial phase; CK_H: CK heating phase; CI_H: CI heating phase; CK_T: CK thermophilic phase; CI_T: CI thermophilic phase; CK_C: CK cooling phase; CI_C: CI cooling phase; CK_M: CK maturation phase; CI_M: CI maturation phase.

## Data Availability

The original contributions presented in this study are included in the article/Supplementary Material. Further inquiries can be directed to the corresponding author.

## Supplementary Materials

The following supporting information can be downloaded at: https://www.mdpi.com/article/10.3390/microorganisms14061177/s1, Figure S1: Abundance dynamics of carbohydrate-active enzymes families involved in lignocellulose degradation at the family level; Figure S2: Correlation dominant carbohydrate-active enzymes families (top nine) with lignocellulosic components based on Spearman correlation analysis; Figure S3: Correlation high-connectivity nodes with lignocellulosic components based on Spearman correlation analysis; Table S1: Abundance of carbohydrate-active enzymes (class level); Table S2: Abundance of carbohydrate-active enzymes related to lignocellulose degradation (according to substrate type); Table S3: The abundance of lignocellulose-degrading microorganisms in different samples; Table S4: Dominant microorganisms (Top 5) in the co-occurrence network at the phylum level; Table S5: High-connectivity node in co-occurrence networks; Table S6: The relative abundance of core taxa in CK and CI across different composting phases; Table S7: Importance ranking of environmental factors with partial Monte Carlo permutation tests.

## Abbreviations

The following abbreviations are used in this manuscript:

ADF	Acid detergent fiber
ADL	Acid detergent lignin
BFGN	Bacterial–fungal co-occurrence network
CAZyme	Carbohydrate-active enzyme
CBMs	Carbohydrate-binding modules
CC	Cellulose content
CDR	Cellulose degradation rate
CEs	Carbohydrate esterases
CI	Thermophilic microbial inoculant
CI_C	CI cooling phase
CI_H	CI heating phase
CI_M	CI maturation phase
CI_T	CI thermophilic phase
CK	Natural composting
CK_C	CK cooling phase
CK_H	CK heating phase
CK_M	CK maturation phase
CK_T	CK thermophilic phase
EC	Electrical conductivity
EM	Effective microorganisms
GHs	Glycoside hydrolases
GTs	Glycosyl transferases
GW	Green waste
GWC	Green waste composting
HC	Hemicellulose content
HDR	Hemicellulose degradation rate
Initial	Initial phase
LC	Lignin content
LDR	Lignin degradation rate
LMGN	Lignocellulose-degrading microorganism network
NDF	Neutral detergent fiber
Pi	Among-module connectivity
RDA	Redundancy analysis
RPKM	Reads per kilobase of transcript per million mapped reads
SynCom	Synthetic microbial community
TKN	Total Kjeldahl nitrogen
TOC	Total organic carbon
WF	*Phanerochaete chrysosporium*
Zi	Within-module connectivity
