# Supplementary material for: Thermophilic Microbial Inoculant Promotes Lignocellulose Degradation During Green Waste Composting
Source: Microorganisms. 2026 May 23;14(6):1177. doi: 10.3390/microorganisms14061177 (PMC13304193; doi:10.3390/microorganisms14061177)
Supplement: Supplementary file 1 [file microorganisms-14-01177-s001.zip › microorganisms-4230643-supplementary.pdf]

Supplementary Materials

of

Thermophilic Microbial Inoculant Promotes Lignocellulose Degradation During Green Waste Composting

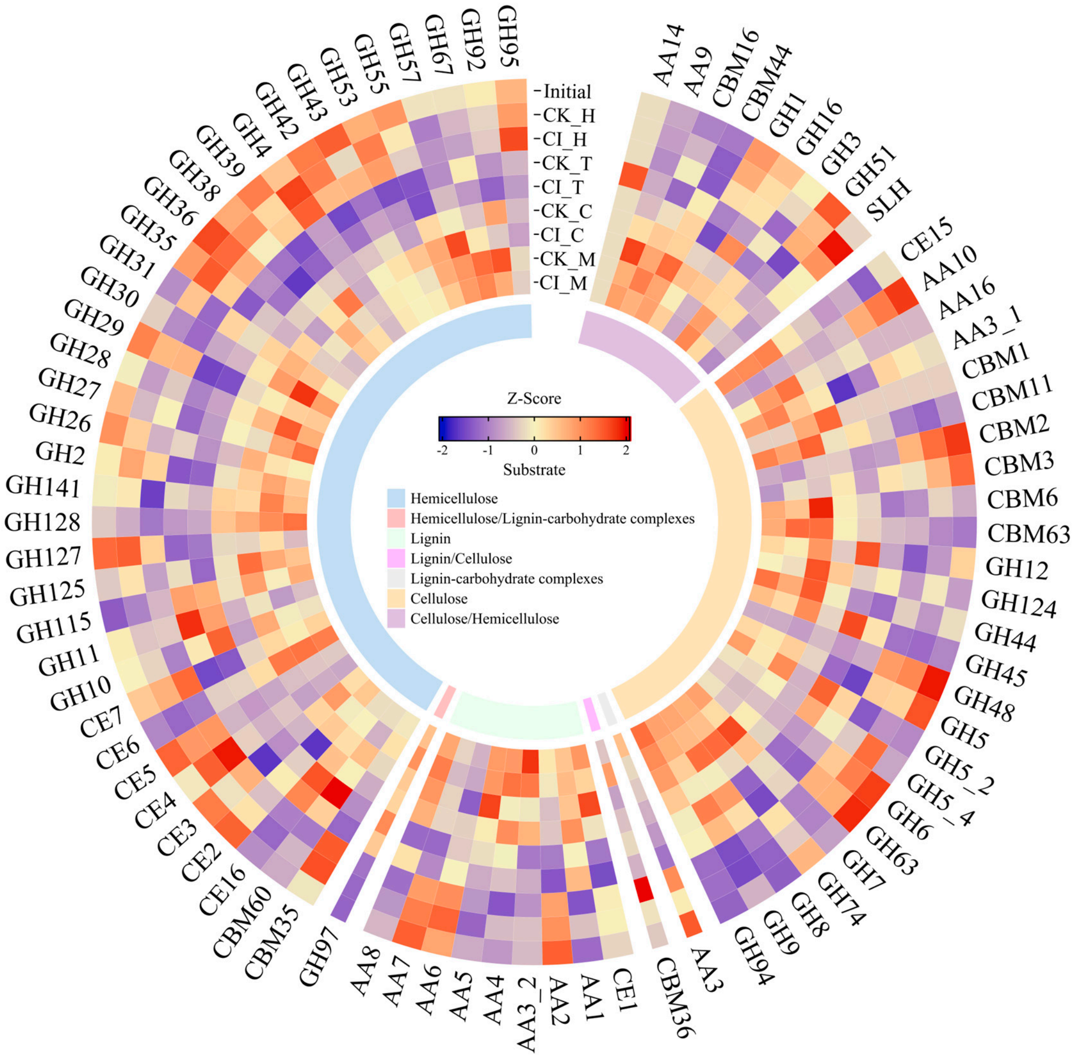

Figure S1. Abundance dynamics of carbohydrate-active enzymes families involved in lignocellulose degradation at the family level. Initial: initial phase; CK_H: CK heating phase; CI_H: CI heating phase; CK_T: CK thermophilic phase; CI_T: CI thermophilic phase; CK_C: CK cooling phase; CI_C: CI cooling phase; CK_M: CK maturation phase; CI_M: CI maturation phase.

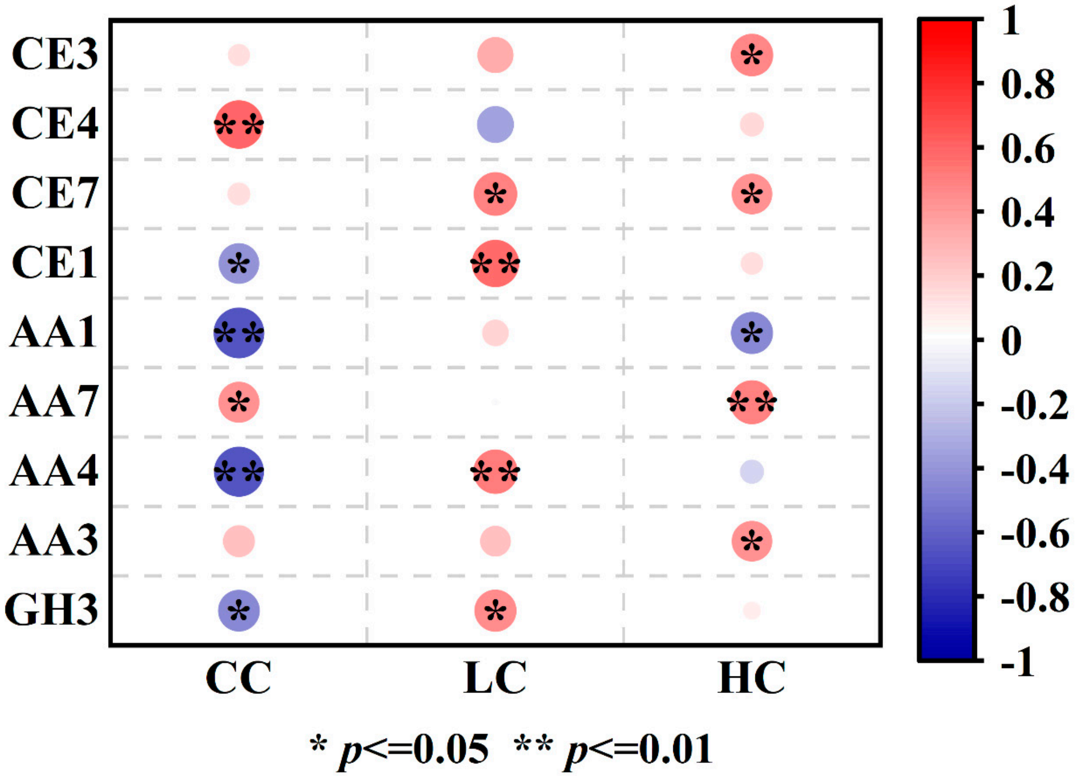

Figure S2. Correlation dominant carbohydrate-active enzymes families (top nine) with lignocellulosic components based on Spearman correlation analysis. HC: hemicellulose content; CC: cellulose content; LC: lignin content.

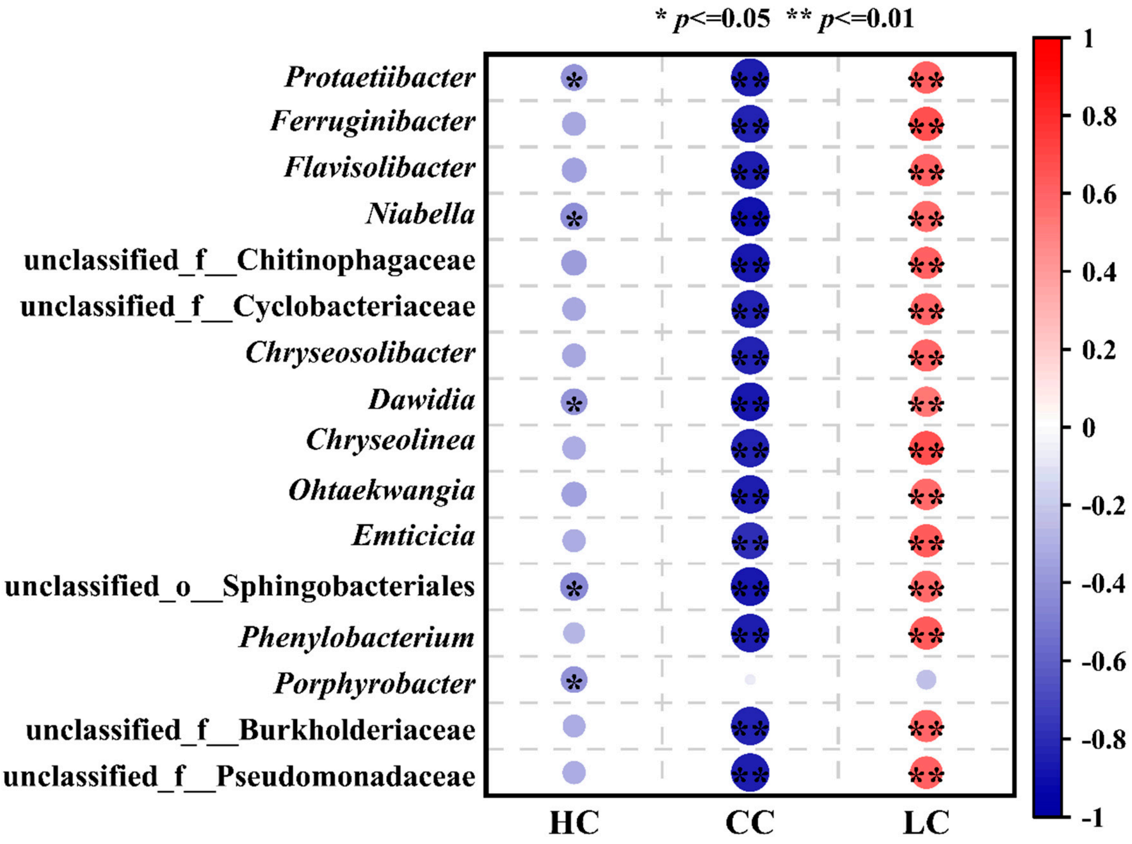

Figure S3. Correlation high-connectivity nodes with lignocellulosic components based on Spearman correlation analysis. HC: hemicellulose content; CC: cellulose content; LC: lignin content.

Table S1. Abundance of carbohydrate-active enzymes (class level).

Treatment	Auxiliary activities	Carbohydrate-binding modules	Carbohydrate esterases	Glycoside hydrolases	Glycosyl transferases	Polysaccharide lyases	S-layer homology
Initial	4837.262	1569.062	9431.241	15064.16	15484.75	1132.617	35.39633
CK_H	4682.983	1541.656	9379.297	14867.51	14128.6	1041.784	90.91607
CI_H	4635.411	1612.537	9677.07	14628.26	14535.97	1116.318	66.0706
CK_T	4271.243	1377.185	8425.996	13884.91	15830.00	970.800	42.98154
CI_T	4225.604	1462.436	8356.298	14166.17	16071.30	1024.029	43.80378
CK_C	4651.669	1676.475	8972.412	15103.27	16988.52	1122.325	27.58627
CI_C	4638.524	1913.738	9211.981	15966.18	18464.48	1338.293	22.39872
CK_M	4765.745	1835.885	9181.392	16016.54	17248.79	1189.538	28.84161
CI_M	4889.398	1837.063	9318.595	16058.02	17602.35	1262.530	18.70478

Initial: initial phase; CK_H: CK heating phase; CI_H: CI heating phase; CK_T: CK thermophilic phase; CI_T: CI thermophilic phase; CK_C: CK cooling phase; CI_C: CI cooling phase; CK_M: CK maturation phase; CI_M: CI maturation phase.

Table S2. Abundance of carbohydrate-active enzymes related to lignocellulose degradation (according to substrate type).

Treatment	Hemicellulose	Lignin	Hemicellulose/Lignin-carbohydrate complexes	Cellulose	Cellulose/Hemicellulose	Lignin/Cellulose	Lignin-carbohydrate complexes
Initial	5666.211	3243.362	2533.75	1864.306	1724.829	1099.632	167.3738
CK_H	5859.31	3232.535	2562.022	1684.491	1733.598	986.0686	125.5879
CI_H	5886.126	3113.223	2563.815	1644.811	1659.306	1062.815	151.8035
CK_T	4862.004	2936.595	2422.455	1522.382	1568.518	889.4065	145.5734
CI_T	4894.936	2876.319	2457.361	1611.507	1582.258	914.2137	160.3283
CK_C	5230.191	3213.243	2546.276	1665.99	1644.554	931.1143	178.6811
CI_C	5475.509	3141.338	2708.788	1917.943	1738.288	950.1535	215.8758
CK_M	5539.889	3256.562	2589.539	1801.241	1696.185	960.8037	213.3758
CI_M	5459.373	3314.955	2643.647	1866.519	1717.379	1021.637	211.4378

Initial: initial phase; CK_H: CK heating phase; CI_H: CI heating phase; CK_T: CK thermophilic phase; CI_T: CI thermophilic phase; CK_C: CK cooling phase; CI_C: CI cooling phase; CK_M: CK maturation phase; CI_M: CI maturation phase.

Table S3. The abundance of lignocellulose-degrading microorganisms in different samples.

Sample Number	Bacteria	Fungi
Initial_1	630500	198
Initial_2	731096	142
Initial_3	586000	186
CK_H_1	687340	116
CK_H_2	630380	118
CK_H_3	504192	90
CI_H_1	544438	206
CI_H_2	645110	140
CI_H_3	540586	68
CK_T_1	555126	340
CK_T_2	665110	222
CK_T_3	649264	210
CI_T_1	601794	158
CI_T_2	650250	332
CI_T_3	625106	276
CK_C_1	649760	550
CK_C_2	653534	618
CK_C_3	634854	630
CI_C_1	676474	902
CI_C_2	659392	1984
CI_C_3	599684	998
CK_M_1	577868	734
CK_M_2	554888	746
CK_M_3	591288	698
CI_M_1	610132	856
CI_M_2	515050	460
CI_M_3	613692	760

Initial: initial phase; CK_H: CK heating phase; CI_H: CI heating phase; CK_T: CK thermophilic phase; CI_T: CI thermophilic phase; CK_C: CK cooling phase; CI_C: CI cooling phase; CK_M: CK maturation phase; CI_M: CI maturation phase.

Table S4. Dominant microorganisms (Top 5) in the co-occurrence network at the phylum level.

Network Name	Microbial taxa and their proportions in the co-occurrence network
Bacterial-Fungal co-occurrence network	Proteobacteria（37.15%），Actinobacteria（19.58%），Firmicutes（10.80%），Bacteroidota（10.52%），Planctomycetes（5.12%）
Lignocellulose-degrading microorganism network	Proteobacteria（34.40%），Bacteroidota（16.35%），Actinobacteria（11.84%），Firmicutes（9.21%），Planctomycetes（4.14%）
Initial	Proteobacteria（34.16%），Bacteroidota（17.36%），Actinobacteria（14.05%），Planctomycetes（5.51%），Firmicutes（5.51%）
CK_H	Proteobacteria（33.62%），Bacteroidota（16.09%），Actinobacteria（14.66%），Firmicutes（9.48%），Planctomycetes（5.46%）
CI_H	Proteobacteria（33.43%），Bacteroidota（18.05%），Actinobacteria（14.79%），Firmicutes（9.17%），Planctomycetes（5.62%）
CK_T	Proteobacteria（34.35%），Bacteroidota（20.78%），Actinobacteria（14.40%），Firmicutes（6.09%），Planctomycetes（4.71%）
CI_T	Proteobacteria（31.75%），Bacteroidota（20.06%），Actinobacteria（14.76%），Firmicutes（5.85%），Planctomycetes（5.01%）
CK_C	Proteobacteria（34.05%），Bacteroidota（19.29%），Actinobacteria（13.33%），Firmicutes（5.00%），Planctomycetes（4.76%）
CI_C	Proteobacteria（34.69%），Bacteroidota（17.91%），Actinobacteria（12.47%），Firmicutes（5.44%），Planctomycetes（4.76%）
CK_M	Proteobacteria（35.38%），Bacteroidota（18.63%），Actinobacteria（12.50%），Firmicutes（4.95%），Planctomycetes（4.95%）
CI_M	Proteobacteria（35.83%），Bacteroidota（18.27%），Actinobacteria（12.65%），Firmicutes（5.15%），Planctomycetes（4.92%）

Initial: initial phase; CK_H: CK heating phase; CI_H: CI heating phase; CK_T: CK thermophilic phase; CI_T: CI thermophilic phase; CK_C: CK cooling phase; CI_C: CI cooling phase; CK_M: CK maturation phase; CI_M: CI maturation phase.

Table S5. High-connectivity node in co-occurrence networks.

**Lignocellulose-degrading microorganism network (33)**
Homoserinibacter; *Protaetiibacte*r; unclassified_f__Microbacteriaceae; unclassified_o__Micrococcales; *Ferruginibacter*; *Flavisolibacter*; *Ginsengibacter*; *Niabella*; unclassified_f__Chitinophagaceae; unclassified_f__Cyclobacteriaceae; *Chryseosolibacter*; *Dawidia*; *Chryseolinea*; *Ohtaekwangia*; *Emticicia*; unclassified_o__Sphingobacteriales; unclassified_p__Candidatus_Andersenbacteria; unclassified_f__Trueperaceae; *Pirellula*; *Phenylobacterium*; *Luteithermobacter*; *Martelella*; *Hyphomicrobium*; *Afipia*; *Mesorhizobium*; unclassified_f__Parvularculaceae; *Porphyrobacter*; *Lautropia*; unclassified_f__Burkholderiaceae; unclassified_f__Pseudomonadaceae; *Opitutus*; unclassified_f__Opitutaceae
**Initial (21)**
Protaetiibacter; *Ferruginibacter*; *Flavisolibacter*; *Niabella*; unclassified_f__Chitinophagaceae; unclassified_f__Cyclobacteriaceae; *Chryseosolibacter*; *Dawidia*; *Chryseolinea*; *Ohtaekwangia*; *Emticicia*; unclassified_o__Sphingobacteriales; unclassified_f__Trueperaceae; *Pirellula*; *Phenylobacterium*; *Afipia*; *Porphyrobacter*; *Lautropia*; unclassified_f__Burkholderiaceae; unclassified_f__Pseudomonadaceae; unclassified_f__Opitutaceae
**CK_H (22)**
Protaetiibacter; *Microlunatus*; *Ferruginibacter*; *Flavisolibacter*; *Niabella*; unclassified_f__Chitinophagaceae; unclassified_f__Cyclobacteriaceae; *Chryseosolibacter*; *Dawidia*; *Chryseolinea*; *Ohtaekwangia*; *Emticicia*; unclassified_o__Sphingobacteriales; unclassified_f__Trueperaceae; *Pirellula*; *Phenylobacterium*; *Porphyrobacter*; *Lautropia*; unclassified_f__Burkholderiaceae; unclassified_f__Pseudomonadaceae; *Rhodanobacter*; unclassified_f__Opitutaceae
**CI_H (20)**
Protaetiibacter; *Ferruginibacter*; *Flavisolibacter*; *Niabella*; unclassified_f__Chitinophagaceae; unclassified_f__Cyclobacteriaceae; *Chryseosolibacter*; *Chryseotalea*; *Dawidia*; *Chryseolinea*; *Ohtaekwangia*; *Emticicia*; unclassified_o__Sphingobacteriales; *Pirellula*; *Phenylobacterium*; *Afipia*; *Porphyrobacter*; unclassified_f__Burkholderiaceae; unclassified_f__Pseudomonadaceae; unclassified_f__Opitutaceae
**CK_T (22)**
Protaetiibacter; *Thermocrispum*; *Ferruginibacter*; *Flavisolibacter*; *Niabella*; unclassified_f__Chitinophagaceae; unclassified_f__Cyclobacteriaceae; *Chryseosolibacter*; *Chryseotalea*; *Dawidia*; *Chryseolinea*; *Ohtaekwangia*; *Emticicia*; unclassified_o__Sphingobacteriales; unclassified_f__Trueperaceae; *Phenylobacterium*; *Hyphomicrobium*; *Afipia*; *Mesorhizobium*; *Porphyrobacter*; unclassified_f__Burkholderiaceae; unclassified_f__Pseudomonadaceae
**CI_T (23)**
Protaetiibacter; *Ferruginibacter*; *Flavisolibacter*; *Niabella*; unclassified_f__Chitinophagaceae;

unclassified_f__Cyclobacteriaceae; *Chryseosolibacter*; *Dawidia*; *Chryseolinea*; *Ohtaekwangia*; *Emticicia*; unclassified_o__Sphingobacteriales; unclassified_f__Trueperaceae; *Pirellula*; *Phenylobacterium*; *Hyphomicrobium*; *Afipia*; *Mesorhizobium*; *Porphyrobacter*; unclassified_f__Burkholderiaceae; *Methylobacter*; unclassified_f__Pseudomonadaceae; *Madurella*
**CK_C (28)**
Protaetiibacter; unclassified_f__Microbacteriaceae; *Ferruginibacter*; *Flavisolibacter*; *Ginsengibacter*; *Niabella*; unclassified_f__Chitinophagaceae; unclassified_f__Cyclobacteriaceae; *Chryseosolibacter*; *Dawidia*; *Chryseolinea*; *Ohtaekwangia*; *Emticicia*; unclassified_o__Sphingobacteriales; unclassified_p__Candidatus_Andersenbacteria; unclassified_f__Trueperaceae; unclassified_c__Ignavibacteria; *Pirellula*; *Phenylobacterium*; *Luteithermobacter*; *Hyphomicrobium*; *Afipia*; *Mesorhizobium*; unclassified_f__Parvularculaceae; *Porphyrobacter*; *Lautropia*; unclassified_f__Burkholderiaceae; unclassified_f__Pseudomonadaceae
**CI_C (32)**
Protaetiibacter; unclassified_f__Microbacteriaceae; unclassified_o__Micrococcales; *Microlunatus*; *Ferruginibacter*; *Flavisolibacter*; *Ginsengibacter*; *Niabella*; unclassified_f__Chitinophagaceae; unclassified_f__Cyclobacteriaceae; *Chryseosolibacter*; *Dawidia*; *Chryseolinea*; *Ohtaekwangia*; *Emticicia*; unclassified_o__Sphingobacteriales; unclassified_p__Bacteroidetes; unclassified_p__Candidatus_Andersenbacteria; unclassified_f__Trueperaceae; *Pirellula*; *Phenylobacterium*; *Luteithermobacter*; *Hyphomicrobium*; *Afipia*; *Mesorhizobium*; g__unclassified_f__Parvularculaceae; *Porphyrobacter*; *Lautropia*; unclassified_f__Burkholderiaceae; unclassified_f__Pseudomonadaceae; unclassified_f__Opitutaceae; *Madurella*
**CK_M (30)**
Protaetiibacter; unclassified_f__Microbacteriaceae; *Ferruginibacter*; *Flavisolibacter*; *Ginsengibacter*; *Niabella*; unclassified_f__Chitinophagaceae; unclassified_f__Cyclobacteriaceae; *Chryseosolibacter*; *Chryseotalea*; *Dawidia*; *Chryseolinea*; *Ohtaekwangia*; *Emticicia*; unclassified_o__Sphingobacteriales; unclassified_p__Candidatus_Andersenbacteria; unclassified_f__Trueperaceae; unclassified_c__Ignavibacteria; *Pirellula*; *Phenylobacterium*; *Luteithermobacter*; *Hyphomicrobium*; *Afipia*; *Mesorhizobium*; unclassified_f__Parvularculaceae; *Porphyrobacter*; *Lautropia*; unclassified_f__Burkholderiaceae; unclassified_f__Pseudomonadaceae; unclassified_f__Opitutaceae
**CI_M (29)**
Protaetiibacter; unclassified_f__Microbacteriaceae; *Ferruginibacter*; *Flavisolibacter*; *Ginsengibacter*; *Niabella*; unclassified_f__Chitinophagaceae; unclassified_f__Cyclobacteriaceae; *Chryseosolibacter*; *Dawidia*; *Chryseolinea*; *Ohtaekwangia*; *Emticicia*; unclassified_o__Sphingobacteriales; unclassified_p__Candidatus_Andersenbacteria; unclassified_f__Trueperaceae; unclassified_c__Ignavibacteria; *Pirellula*; *Phenylobacterium*; *Luteithermobacter*; *Hyphomicrobium*; *Afipia*; *Mesorhizobium*; unclassified_f__Parvularculaceae; *Porphyrobacter*; *Lautropia*; unclassified_f__Burkholderiaceae; unclassified_f__Pseudomonadaceae; unclassified_f__Opitutaceae
**Shared high-connectivity nodes in the lignocellulose-degrading microorganism network and its**

**subnetworks (16)**
Protaetiibacter; *Ferruginibacter*; *Flavisolibacter*; *Niabella*; unclassified_f__Chitinophagaceae; unclassified_f__Cyclobacteriaceae; *Chryseosolibacter*; *Dawidia*; *Chryseolinea*; *Ohtaekwangia*; *Emticicia*; unclassified_o__Sphingobacteriales; *Phenylobacterium*; *Porphyrobacter*; unclassified_f__Burkholderiaceae; unclassified_f__Pseudomonadaceae

The numbers in parentheses represent the count of high-connectivity node. Initial: initial phase; CK_H: CK heating phase; CI_H: CI heating phase; CK_T: CK thermophilic phase; CI_T: CI thermophilic phase; CK_C: CK cooling phase; CI_C: CI cooling phase; CK_M: CK maturation phase; CI_M: CI maturation phase.

Table S6. The relative abundance of core taxa in CK and CI across different composting phases.

**Treatment**	***Porphyrobacter***	***Chryseolinea***	***Protaetiibacter***	**unclassified_f__Burkholderiaceae**
Initial	2.58E-06	9.31E-07	6.36E-07	3.74E-06
CK_H	1.53E-05	1.06E-07	1.70E-06	2.92E-06
CI_H	7.48E-06	7.03E-07	1.69E-06	2.39E-06
CK_T	5.13E-05	2.19E-06	7.51E-06	9.07E-06
CI_T	3.01E-05	3.00E-06	7.19E-06	1.11E-05
CK_C	2.04E-05	7.99E-06	4.12E-05	2.25E-05
CI_C	7.19E-06	1.56E-05	2.70E-05	2.30E-05
CK_M	1.85E-05	1.82E-05	5.70E-05	3.07E-05
CI_M	8.77E-06	1.51E-05	6.48E-05	4.16E-05

Initial: initial phase; CK_H: CK heating phase; CI_H: CI heating phase; CK_T: CK thermophilic phase; CI_T: CI thermophilic phase; CK_C: CK cooling phase; CI_C: CI cooling phase; CK_M: CK maturation phase; CI_M: CI maturation phase.

Table S7. Importance ranking of environmental factors with partial Monte Carlo permutation tests.

Biological factors	Indicator	Explained variation (%)	Contribution (%)	*F*-value	*p*-value
Carbonhydrate-active Enzymes（Class level)	CC	55.3	65.4	30.9	0.002
	Temperature	16.4	19.4	14	0.002
	pH	4.6	5.5	4.5	0.018
	HC	3.8	4.5	4.2	0.020
	TOC	1.8	2.2	2.1	0.122
	EC	1.4	1.6	1.6	0.194
	TN	1.1	1.3	1.3	0.254
	LC	<0.1	<0.1	<0.1	0.974
Key carbonhydrate-active Enzymes Families	Temperature	18.6	28.5	5.7	0.010
	pH	16.6	25.5	6.1	0.008
	TOC	13.1	20.1	5.8	0.004
	EC	10.4	16	5.6	0.006
	CC	2.1	3.2	1.1	0.334
	TN	1.9	3	1.0	0.344
	HC	1.5	2.4	0.8	0.424
	LC	0.9	1.4	0.5	0.650
Core taxa	LC	47.3	56.9	22.4	0.002
	CC	18.3	22	12.7	0.002
	TN	6.7	8.1	5.6	0.006
	Temperature	4.5	5.5	4.3	0.016
	TOC	2.6	3.1	2.6	0.068
	EC	1.9	2.3	2.1	0.138
	pH	1.2	1.4	1.3	0.278
	HC	0.6	0.7	0.6	0.594

EC: electrical conductivity; TOC: total organic carbon; TKN: total Kjeldahl nitrogen; HC: hemicellulose content; CC: cellulose content; LC: lignin content.
